# Comparative Physiology of Oleaginous Species from the *Yarrowia* Clade

**DOI:** 10.1371/journal.pone.0063356

**Published:** 2013-05-07

**Authors:** Stéphanie Michely, Claude Gaillardin, Jean-Marc Nicaud, Cécile Neuvéglise

**Affiliations:** 1 INRA, UMR 1319 Micalis, Jouy-en-Josas, France; 2 AgroParisTech, Micalis, Jouy-en-Josas, France; 3 CNRS, Micalis, Jouy-en-Josas, France; University of Nebraska, United States of America

## Abstract

*Yarrowia lipolytica* is a genetically tractable yeast species that has become an attractive model for analyses of lipid metabolism, due to its oleaginous nature. We investigated the regulation and evolution of lipid metabolism in non-Saccharomycetaceae yeasts, by carrying out a comparative physiological analysis of eight species recently assigned to the *Yarrowia* clade: *Candida alimentaria, Y. deformans, C. galli, C. hispaniensis, C. hollandica, C. oslonensis, C. phangngensis* and *Y. yakushimensis*. We compared the abilities of type strains of these species to grow on 31 non hydrophobic (sugars and other carbohydrate compounds) and 13 hydrophobic (triglycerides, alkanes and free fatty acids) carbon sources. Limited phenotypic diversity was observed in terms of the range of substrates used and, in the case of short-chain fatty acids, their toxicity. We assessed the oleaginous nature of these species, by evaluating their ability to store and to synthesize lipids. The mean lipid content of cells grown on oleic acid differed considerably between species, ranging from 30% of cell dry weight in *C. oslonensis* to 67% in *C. hispaniensis*. Lipid synthesis in cells grown on glucose resulted in the accumulation of C18:1 (n-9) as the major compound in most species, except for *C. alimentaria* and *Y. yakushimensis*, which accumulated principally C18:2(n-6), and *C. hispaniensis*, which accumulated both C16:0 and C18:1(n-9). Thus, all species of the clade were oleaginous, but they presented specific patterns of growth, lipid synthesis and storage, and therefore constitute good models for the comparative analysis of lipid metabolism in this basal yeast clade.

## Introduction

Some yeast species can store and synthesize lipids from different carbon sources. Yeast species are described as “oleaginous” if the lipids they accumulate account for more than 20- to 25% of their biomass [1]. Oleaginous yeasts are dispersed throughout the entire phylogenetic tree of basidiomycetes and hemiascomycetes. The hemiascomycetous yeast *Yarrowia lipolytica* has emerged as an important model for lipid metabolism studies. Like other oleaginous yeasts, it can grow on sugars, such as glucose [2], [3], and on hydrophobic substrates (HS) [4]. It can also synthesize and store lipids [5]. In addition, *Y. lipolytica* is highly tractable genetically, making it a good model species for biotechnological applications, particularly for single-cell oil production [6], [7], [8]. However, the amount of lipid that accumulates depends on the strain, the carbon source and growth conditions. Under optimal conditions, some wild strains of *Y. lipolytica* can store 36 % of their cell dry weight (CDW) as lipids [1]; similar levels are observed in fed-batch cultures with glucose/glycerol [9]; 43% of the CDW may be lipid in continuous fermentations of industrial glycerol [10] and up to 54% may be lipid in batch cultures on a stearin-based medium [11], [12]. However, in flask cultures in which nitrogen concentration is not controlled, wild strains of *Y. lipolytica* do not generally accumulate more than 15% of their CDW as lipids when grown in glucose medium [13], [14], [15] or in wastewater [14], [15].

Most of the lipids accumulating in *Y. lipolytica* are triacylglycerols rather than free fatty acids (FFA), the ratio of these two types of compounds being 5/1 (triacylglycerols/FFA) [16]. C16 and C18 compounds are the most abundant lipids stored by this yeast. However, their relative quantities depend on the growth medium used. The *Y. lipolytica* strain W29 ( = CBS 7405) stores mostly C18:1 (54%), C16:0 (26%), C18:2 (12%) and a little C16:1 when cultured on glucose, whereas it accumulates C18:1 (66%), C16:1 (16%), C18:2 (9%) and a little C16:0 when cultured on oleic acid [17].

We investigated the emergence of oleaginous properties in yeasts, by comparative studies of *Y. lipolytica* W29 and strains from the eight species recently identified as members of the *Yarrowia* clade: *Candida (C.) alimentaria, Y. deformans, C. galli, C. hispaniensis, C. hollandica, C. oslonensis, C. phangngensis* and *Y. yakushimensis* [18], [19], [20], [21], [22], [23]. These strains were isolated from various biotic and abiotic environments. Limited physiological data for these strains are available, mostly based on the assimilation tests used for their identification. All these strains are non fermentative, grow as a mixture of cells and hyphae and use a very limited range of sugars, mostly glucose, as carbon sources. Additional data for growth on other carbon sources have provided evidence of phenotypic diversity [24], although different tests were carried out on different strains. For example, *C. hispaniensis* and *C. oslonensis* have been reported to use galactose and sorbose, which are only weakly metabolized, if at all, by other species, whereas *C. hispaniensis* is the only one of the species considered able to make use of trehalose. *C. galli* is the only one of these species that has been reported to grow in a vitamin-free environment; the failure of the other species to do so may result from thiamin auxotrophy, as reported in *Y. lipolytica* [25]. Tolerance to 10% NaCl differs between species, as does maximum growth temperature, which ranges from 27°C for *C. alimentaria* to 37°C for *C. phangngensis*, with most strains of other species growing little at temperatures above 30 to 32°C. It remains unclear whether this phenotypic diversity extends to the metabolism of hydrophobic compounds and, more generally, whether the oleaginous character of *Y. lipolytica* is particular to this species or common to some or all members of its clade. For example, data for growth on hexadecane and lipase production are patchy or absent for these species, with the exception of *Y. deformans*, in which three lipases have been purified and the corresponding genes have been cloned and sequenced [26], [27], [28].

We thus investigated the capacities of the type strains of the eight species and of the French wild-type strain W29 to use various hydrophobic and non hydrophobic substrates as carbon sources. Detailed growth kinetics were obtained on glucose and oleic acid media. Lipid profiles were determined, together with the capacity to accumulate and to synthesize lipids. The knowledge about the physiology of these strains provided by this study opens up new possibilities for genetic and transcriptomic analyses within the *Y. lipolytica* clade. The long-term objective will be to obtain a full understanding of lipid metabolism in this group, to improve the suitability of *Y. lipolytica* as a tool for biotechnological applications.

## Materials and Methods

### Yeast strains, media and growth conditions

The strains of the *Yarrowia* clade investigated in this study, their origins and references are listed in Table 1. All are wild-type prototroph strains. Strain names are abbreviated as follows: *YALI* (*Y. lipolytica* W29), *YAYA* (*Y. yakushimensis* CBS10253), *YADE (Y. deformans* CBS2071), *YAGA (C. galli* CBS9722), *YAOS (C. oslonensis* CBS10146), *YAHO (C. hollandica* CBS4855), *YAPH (C. phangngensis* CBS10407), *YAAL (C. alimentaria* CBS10151), *YAHI (C. hispaniensis* CBS9996). All strains were cultured at 28°C, with the exception of *YAAL*, for which the optimal growth temperature was 21°C (Additional Figure S1). Minimal medium base (MMB) contained 0.17% (wt/vol) yeast nitrogen base without amino acids and ammonium sulfate (Difco, Paris, France), 0.5% (wt/vol) NH_4_Cl and 50 mM phosphate buffer pH 6.8; 1.5% (wt/vol) agar was added when necessary. The solid rich medium base (YP) contained 1% (wt/vol) yeast extract and 1% (wt/vol) peptone, together with 1.5% (wt/vol) agar. Carbon sources were added at a final concentration of 2%; hydrophobic substrates were first emulsified by sonication of a 20% mixture in the presence of 0.625% Tween 40.

**Table 1:** Characteristics of the strains used in this study.

Species	Strain number	Abbreviation	Place of isolation	Country	Reference
*Yarrowia lipolytica*	CBS 7504 = W29	*YALI*	Parisian sewer	France	[25]
*Yarrowia deformans*	CBS 2071	*YADE*	Fingernail	Austria	[23]
*Candida galli*	CBS 9722	*YAGA*	Chicken liver	Georgia, USA	[18]
*Yarrowia yakushimensis*	CBS 10253	*YAYA*	Termite gut	Japan	[23]
*Candida oslonensis*	CBS 10146	*YAOS*	Kiwifruit yogurt	Norway	[20]
*Candida phangngensis*	CBS 10407	*YAPH*	Seawater	Thailand	[21]
*Candida hollandica*	CBS 4855	*YAHO*	Back of a cow	The Netherlands	[20]
*Candida alimentaria*	CBS 10151	*YAAL*	Cured ham	Norway	[20]
*Candida hispaniensis*	CBS 9996 = Y−5580	*YAHI*	*Spondylus buprestoides* larva	Spain	[19]

Abbreviations: CBS, Centraalbureau voor Schimmelcultures, Utrecht, The Netherlands;  = W29, other common name used;  = Y−5580, name from the NRRL collection.

### Growth tests

Drop tests were performed with the 13 hydrophobic substrates (HS) listed in Table 2. Both solid rich medium and solid minimal medium were supplemented with 2% emulsified hydrophobic substrates, with the exception of alkanes, for which a paper inserted into the plate lid was soaked with alkane daily, to offset the effects of evaporation. Precultures were grown on plates of minimal medium containing 0.17% (wt/vol) yeast nitrogen base without amino acids and ammonium sulfate (Difco, Paris, France), 0.5% (wt/vol) (NH_4_)_2_SO_4_ and 1% glucose. We plated 3 µl of each of a set of five-fold dilutions, corresponding to 2 to 1250 cells. Pictures were taken daily or every two days over a period of three weeks. Growth was considered to be delayed, weak or slow with respect to the strain with the best growth in the study [29]. API ID 32 C galleries (Biomérieux, France) of 32 cupules (31 different carbon substrates plus one control), were used to evaluate the assimilation of a set of carbon sources (Additional Table S1).

**Table 2:** List of hydrophobic substrates used.

Class of hydrophobic substrate	Substrate	Number of carbon atoms and insaturation number
**Triglycerides**	tributyrin	C4
	triolein	C18:1
**Methylates**	methyl hexanoate	C6
	methyl decanoate	C10
	methyl myristate	C14
	methyl palmitate	C16
	methyl oleate	C18:1
**Free fatty acids**	hexanoic acid	C6
	oleic acid	C18:1
	erucic acid	C22:1
**Alkanes**	decane	C10
	dodecane	C12
	hexadecane	C16

### Microtiter plate culture analysis

Growth tests were performed in 100 µl cultures in 96-well plates, with constant shaking, in the presence of 2% arabinose, fructose, glucose, N-acetyl-glucosamine, ribose, saccharose or xylose as the carbon source. Precultures were grown on minimal medium plates, as for the growth tests. Growth was monitored by measuring the optical density at 600 nm (OD_600_ nm) at 10-minute intervals, with a microtiter plate reader (Biotek, Colmar, France). For each strain and set of conditions, we used two biological replicates.

### Growth curves and parameter determination

The nine strains were grown in MMB plus 0.15% yeast extract, with 2% glucose or 2% oleic acid, in 500-ml Erlenmeyer baffled flasks, to improve the dispersion of alkanes and oxygen supply [30]. Cells from overnight YPD cultures were used to inoculate the culture at an initial OD_600_ of 0.5. Biomass production was followed by measuring OD_600nm_ every two or three hours. Cells grown in the presence of oleic acid were washed twice with 0.5% bovine serum albumin and then once with 0.9% NaCl before OD determination.

We used a custom-developed R software script [31] to derive parameters from the growth curves: length of the lag phase λ, growth rate corresponding to the maximum slope (µmax) and maximum cell density.

### Lipid analysis

For fatty acid (FA) determinations, the strains were precultured and cultured under the same conditions and in the same media as for growth curve experiments. Two biological replicates were carried out. Samples of yeast cells were taken at three different growth stages and centrifuged at 3000 g for 5 min. The cell pellets were washed once with a half volume of 0.9% NaCl for glucose conditions and twice with a half volume of 0.5% bovine serum albumin and then once with a half volume of 0.9% NaCl for oleic acid conditions. Washed cells were freeze-dried for 24 h at 80°C.

The FA from the equivalent of 10 OD_600_ units of freeze-dried harvested cells were converted into their methyl esters by the Browse method [32]. The FA methyl esters were analyzed by gas chromatography, with a Varian 430-GC instrument equipped with a flame ionization detector and a CP SIL SCB low Bleed/MS column, for which the bleed specification at 260°C was 3 pA (30 m, 0.25 mm, 0,25 µm). FA were identified by comparison with commercial FA methyl ester standards (FAME32; Supelco) and quantified by the internal standard method, involving the addition of 75 µg of commercial C17:0 (Sigma). We determined the FA compositions from chromatograms with custom-developed Python and R software scripts [31].

### Phylogeny

The phylogenetic tree was constructed by the concatenation of seven markers known to be representative of species evolution in fungi [33]. These markers were MS277 (YALI0B08756g homologs), MS444 (YALI0A00264g homologs), MS456 (YALI0B18722g homologs), MS561 (YALI0B16434g homologs), FG598 (YALI0A02695g homologs), and FG610 (YALI0D11220g homologs), together with EF-1alpha (YALI0C09141g homologs). DNA was extracted as described by Hoffman and Winston [34]. PCR amplifications were performed with an Eppendorf 2720 thermocycler, ex-*Taq* polymerase (Takara) and the primers listed in Additional Table S2. Both strands were sequenced by GATC Biotech (Mulhouse, France).

The Staden package was used to analyze sequencing reads [35]. We used MUSCLE [36] to align sequences, and columns of gap-containing residues were removed manually. A final alignment of 2204 amino acids was obtained after sequence concatenation. Phylogenetic trees were constructed either by neighbor-Joining in ClustalX [37], or by maximum likelihood, with PHYML [38] and a JTT substitution model corrected for heterogeneity between sites by a Γ-law distribution, with four different categories of evolution rates. The proportion of invariable sites and the α-parameter of the Γ-law distribution were optimized according to the data. A bootstrap value was calculated from 100 replicates.

## Results

### Use of non hydrophobic carbon sources

Carbon assimilation tests have been performed for identification of the different species of the *Yarrowia* clade [18], [19], [20], [21], [22], [23]. However, to obtain a homogeneous set of data, we assessed the ability of the nine strains to use 31 compounds. API galleries were initially used, for 30 compounds. The results were compared with the data available from the CBS (http://www.cbs.knaw.nl/). For 11 carbon sources, our observations were not consistent with those of the CBS for some strains, possibly reflecting differences in experimental procedures (API galleries, microtiter plate or agar plate tests) or carbon source concentration. Thus, liquid cultures in microtiter plates were used for these substrates, with glucose and sucrose used as positive and negative controls, respectively. These experiments confirmed that *YALI* and *YAPH* did not grow on arabinose, that *YAGA* grew on glycerol, and that *YALI* and *YADE* grew on N-acetyl-glucosamide. We also tested fructose as a carbon source under the same conditions (Additional Table S1).

Finally, only three of the 31 compounds (fructose, glycerol and glucose) supported the growth of all species and none of the species could grow on 21 other carbon sources. For the remaining seven compounds, differences in growth were observed between species. *YAHI*, which belongs to the most divergent species, was unique in being able to grow on trehalose and unable to use lactate and erythritol. Growth on mannitol, N-acetyl-glucosamine, potassium gluconate and sorbitol varied between strains.

### Growth capacities on hydrophobic substrates

**Figure 1:**
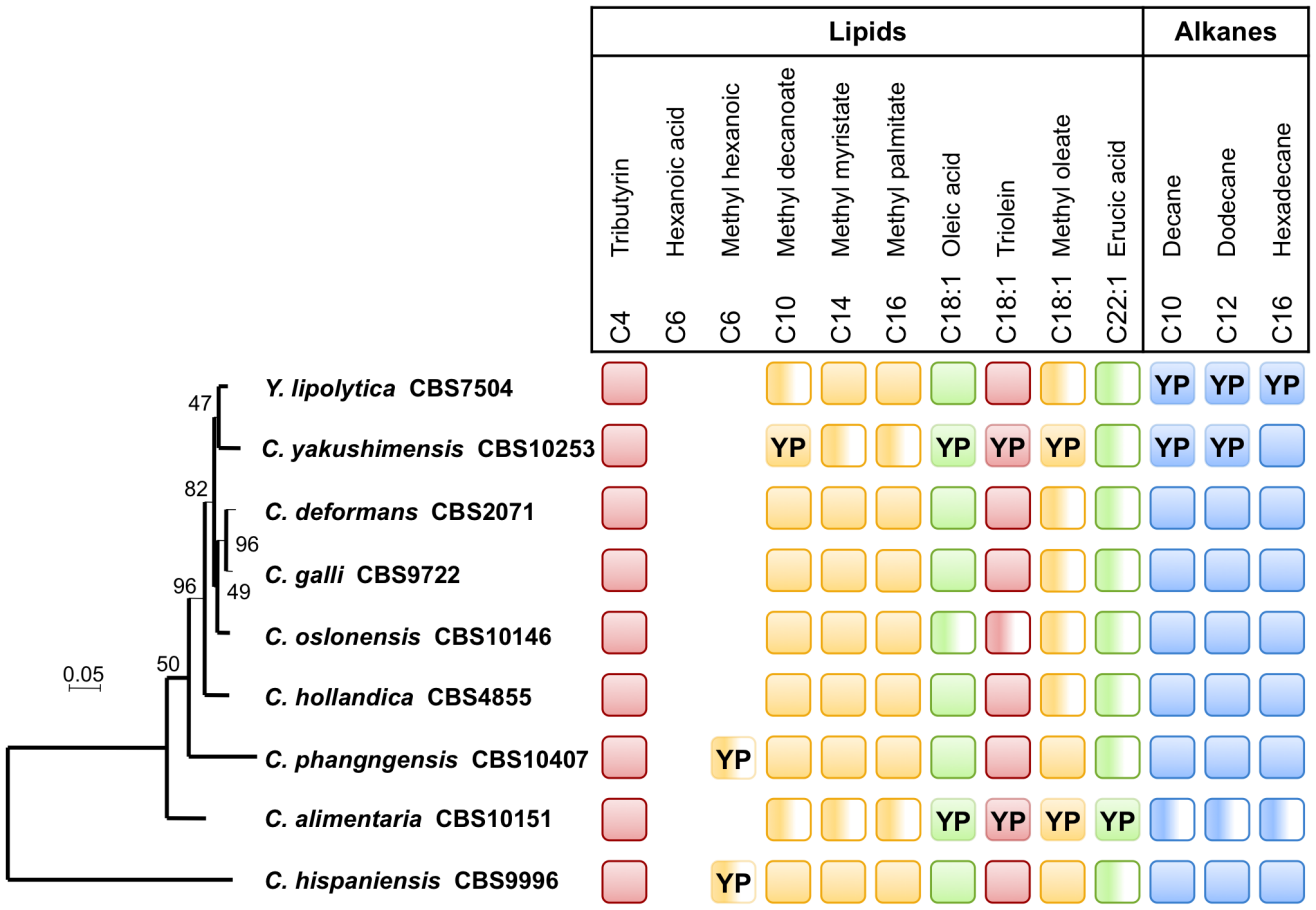
Growth capacities of *Yarrowia* strains on 13 hydrophobic substrates as a function of their phylogenetic position. Squares represent growth capacity on minimal and rich (YP) media supplemented with triglycerides in red, methylates in yellow, free fatty acids in green and alkanes in blue. The squares marked YP reflect the capacity to grow on the YP base used as a carbon source, in the presence of the corresponding HS. Half-colored squares indicate slow or weak growth. The absence of a square indicates an absence of growth on both YP and minimal media. The phylogenetic tree was constructed by the concatenation of seven markers (2204 amino acids) and its robustness was estimated with a bootstrap of 100 replicates.

We assessed growth on lipids and alkanes by carrying out drop tests for 13 HS, initially on MMB (Table 2). Three of the 10 lipid sources tested supported the growth of all species (tributyrin, methyl myristate and methyl palmitate), two were not used by any of the species (hexanoic acid and methyl hexanoic, both C6 compounds) and the remaining five were used by some, but not all species. The strains differed in their ability to grow on the three alkanes (Figure 1). Thus, all strains were able to grow on at least some hydrophobic substrates. However, some displayed growth delay or an absence of growth on specific substrates. *YAAL* could not grow on C18:1 whatever the type of compound supplied (triglyceride, methyl or fatty acid) or on C22:1. On all other media, growth was delayed or weak, except in the presence of tributyrin. Similarly, *YAYA* did not grow on the three types of C18:1 or on C10 (methyl decanoate). On all other lipids except tributyrin, growth was delayed or weak and hexadecane (C16) was the only alkane of the three tested that supported the growth of this strain. Surprisingly, *YALI* formed no colonies on alkane minimal media under these growth conditions.

**Figure 2:**
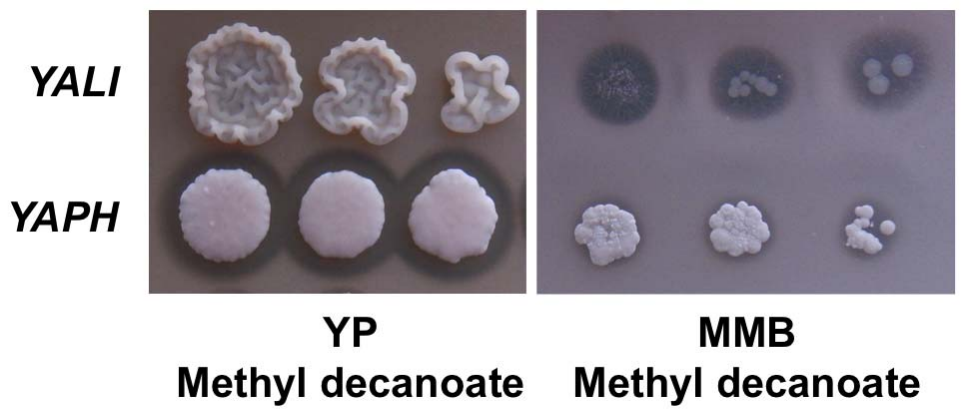
Drop tests on methyl decanoate in MMB and YP media, after 9 days of culture. Three spots are shown, corresponding to approximately 25 to 625 cells, from right to left. Strains are represented by an abbreviation of their species name, as shown in Table 1.

**Figure 3:**
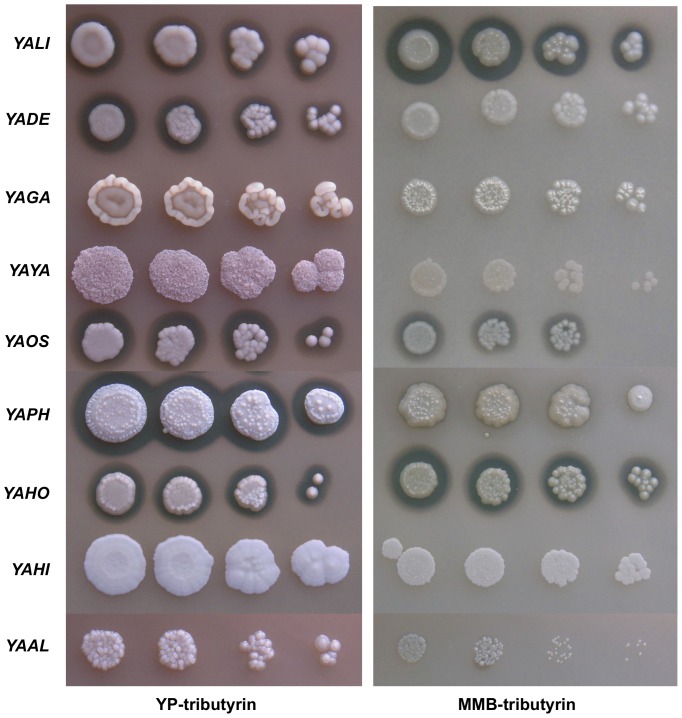
Drop tests on YP- and MMB-tributyrin media after 5 days of culture. Four spots are shown, with approximately 5 to 625 cells, from right to left. A halo is present around strains *YALI*, *YADE*, *YAOS*, *YAPH* and *YAHO*. Strains are represented by an abbreviation of their species name, as shown in Table 1.

There are three possible reasons for the inability of a strain to grow on a given substrate: the substrate may not be taken up by the cells, it may not be metabolized or it may have a toxic effect, preventing growth. We tested the hypothesis of toxicity by repeating the drop tests on a rich medium (YP, yeast extract plus peptone) supplemented with hydrophobic compounds (Figure 2, Figure 3 and additional Figure S2). Under these conditions, all strains could grow in the presence of all the hydrophobic substrates tested, with two exceptions, as described below. The results of these tests indicated that some hydrophobic substrates neither supported nor inhibited cell growth. The two exceptions were the two C6 compounds (hexanoic acid and methyl hexanoic acid), which strongly inhibited all strains, although *YAPH* and *YAHI* were able to grow on YP-methyl hexanoic after 20 days. Similarly, on MMB-methyl decanoate, no growth defect was observed at low cell density, whereas growth was strongly inhibited at high cell density. No such growth inhibition was observed on YP-methyl decanoate (Figure 2).

Morphology on plates was very variable, ranging from smooth to rough colonies, and from superficial to agar-invasive forms, depending on the medium (see figures 2, 3 and data not shown). The presence of a halo around cells grown on plate containing insoluble substrates generally reflects the secretion of surfactants or of extracellular enzymes, such as lipases or esterases [39]. Only four of the 13 HS tested on MMB and YP media were associated with halo formation: tributyrin, methyl decanoate, oleic acid and triolein (Additional Table S3). Some strains formed a halo on MMB or YP tributyrin plates, whereas others did not (Figure 3). The presence of a halo was not associated with colony size and depended on the medium, *i.e.* MMB or YP. For instance, *YAOS* formed a large halo on YP-tributyrin but grew less well than *YAHI*, which did not form a halo.

### Lipid storage capacities are variable within the *Yarrowia* clade

The best way to determine the storage capacity of yeasts with minimal bias due to *de novo* synthesis is to estimate their lipid content on a lipid-based growth medium. We determined growth characteristics, such as lag phase, generation time and maximal OD_600_, on the standard medium used for this purpose in *Y. lipolytica*: an oleic acid medium supplemented with small amounts of yeast extract to facilitate the transition from sugar to hydrophobic carbon sources [17]. (Figure S3A). All strains grew in this medium, without a lag phase (Table 3). Generation time and maximum cell growth differed between species, ranging from 2.06 h to 3.91 h and from 17.6 to 53.1 OD_600_, respectively. *YAHI* was the fastest growing species on oleic acid, reaching 53.10 OD_600_ units after about 23 h of growth, with a generation time of 2.06 h. By contrast, *YAGA* seemed be assimilate oleic acid much less efficiently, with a generation time almost twice that of *YAHI*. We were therefore unable to use the standard protocols for estimating lipid storage in *Y. lipolytica*. Thus, to ensure that the data obtained were comparable, we had to identify similar physiological stages not necessarily corresponding to the same time in culture.

**Table 3:** Growth parameters on oleic acid and glucose media.

Strain	µmax (h^−1^)	Generation time (h)	Lag phase λ (h)	Maximum cell density (OD_600_)
**Oleic acid**				
*YALI*	0.21	3.27	-	35.31
*YAYA*	0.18	3.73	-	20.35
*YADE*	0.25	2.74	-	17.60
*YAGA*	0.18	3.91	-	23.52
*YAOS*	0.22	3.12	-	19.98
*YAHO*	0.26	2.67	-	33.66
*YAPH*	0.32	2.18	-	46.8
*YAAL*	0.28	2.46	-	28.27
*YAHI*	0.34	2.06	-	53.10
**Glucose**				
*YALI*	0.44	1.56	2.54	25.00
*YAYA*	0.71	0.97	5.19	20.86
*YADE*	0.78	0.89	5.61	17.80
*YAGA*	0.42	1.64	3.38	23.71
*YAOS*	0.63	1.09	5.71	19.00
*YAHO*	0.53	1.32	3.85	27.86
*YAPH*	0.57	1.21	1.26	29.14
*YAAL*	0.64	1.08	8.77	23.2
*YAHI*	0.53	1.32	7.93	17.3

We estimated the maximal level of lipid storage, by determining cellular lipid content at various time points. Based on the growth curves obtained, we selected three time points between the beginning of the exponential phase and the deceleration phase. These time points corresponded roughly to the time required to obtain one fifth of the maximum OD_600_ (OD max), half the OD max and the OD max. An example is provided in Additional Figure S4. For lipid extraction, we collected cells for two biological replicates at about the three time points derived from the growth curve (Additional Table S4).

**Figure 4:**
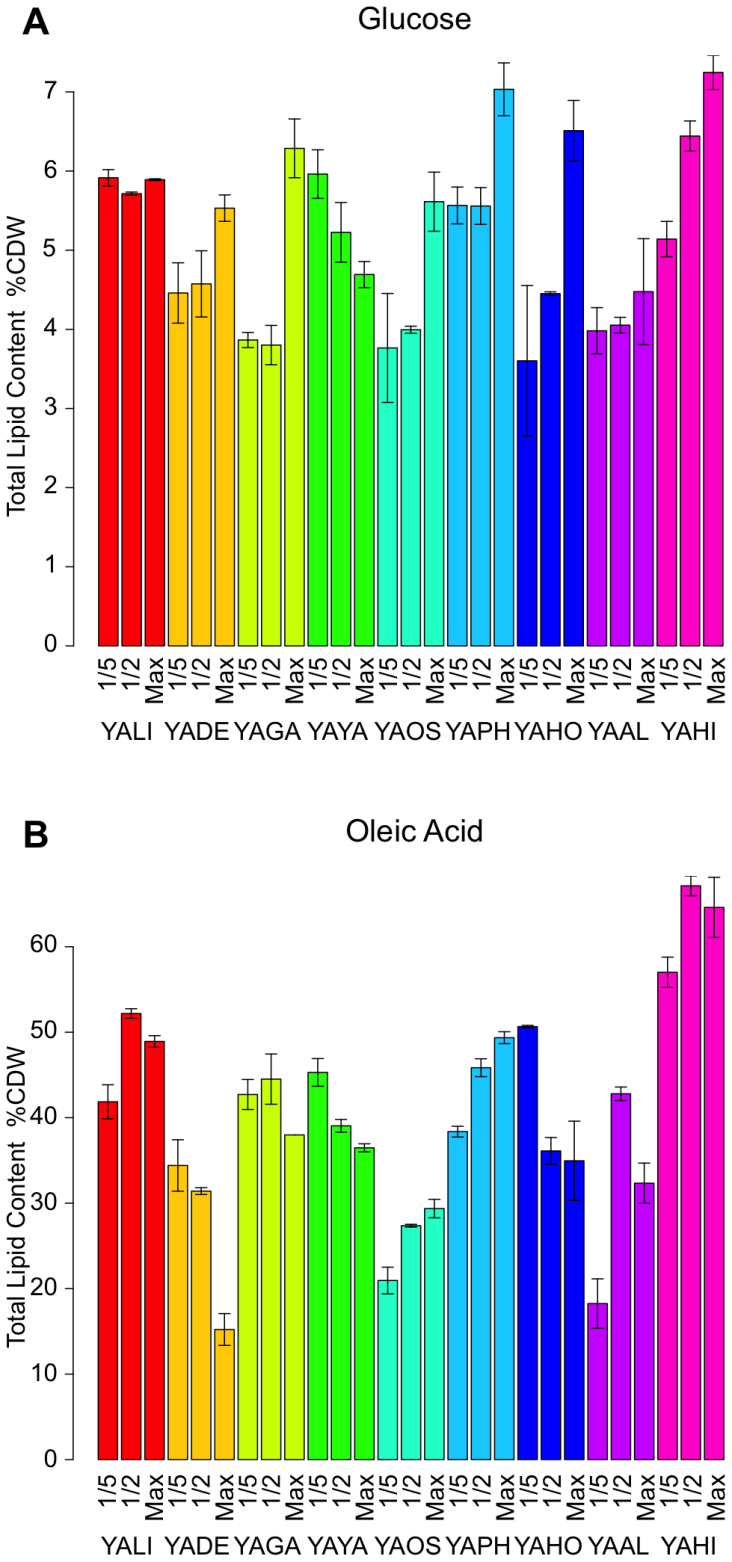
Lipid content (% CDW) of *Yarrowia* strains over time. For each species, the histogram represents three different physiological states: 1/5 of maximum growth, 1/2 maximum growth and maximum growth. Error bars indicate the deviation from the mean deduced from two replicates. Analyses were performed on both glucose (A) and oleic acid (B). Strains are represented by an abbreviation of their species name, as shown in Table 1.

The total lipid content of cells grown on oleic acid is shown in Figure 4A. Three different types of behavior were observed. The first was characterized by an increase in lipid accumulation during the exponential growth phase followed by a decrease in lipid levels during the deceleration phase, possibly corresponding to lipid reconsumption. This pattern was observed in *YALI*, *YAGA*, *YAAL* and *YAHI*. The second type of pattern consisted of a continuous increase in lipid content and was observed in *YAOS* and *YAPH*. The three remaining strains, *YADE*, *YAYA* and *YAHO*, displayed decreases in lipid content over time. For instance, the lipid content of *Y. deformans* CBS2071 decreased from 34.4% to 15.2% of CDW.

The maximal level of lipid storage differed considerably between species, ranging from 29.4% of CDW for *YAOS* to 67.1% of CDW for *YAHI*. *YALI* was the second most efficient strain for lipid storage, which accounted for 52.2% of CDW.

By contrast, overall lipid composition remained stable over time for all species (Additional Table S5). Most of the lipid was in the form of C18:1(n-9), as expected given that the main carbon source in the growth medium was oleic acid. In *YALI*, *YAGA*, *YAHI* and *YAHO*, the amount of C18:1(n-9) varied from 23.1 to 38.3 % of CDW, suggesting that oleic acid uptake was more efficient in these species than in the other strains tested (Additional Fig S5). The second most abundant compound accumulating was generally C16:1(n-7), but its level was always low and varied from 0.7 to 11.8 % of CDW.

### *De novo* lipid synthesis

We then determined the capacity for *de novo* lipid synthesis of the nine strains. This capacity was estimated on a glucose medium supplemented with yeast extract: a rich medium devoid of hydrophobic compounds. As in evaluations of storage capacity, we first established growth curves (Additional Figure S3B). By contrast to what was observed on oleic acid, there was a conspicuous lag phase for all strains, lasting 1.26 to 8.77 h, and generation time was clearly shorter than on oleic acid media, but remained variable (0.89 to 1.64 h). OD max values were similar on the two media for all strains except *YALI*, *YAPH* and *YAHI*, for which the OD max on glucose was lower than that on oleic acid, by a factor of up to three. For instance, the OD max for *YAHI* was 17.3 on glucose and 53.1 on oleic acid.

We then measured lipid contents at three time points defined as described above: 1/5, 1/2 and 1 OD max (Additional Table S4). Almost all the strains presented similar patterns of total lipid content, with large increases during the deceleration phase (Figure 4B). However, slight differences in kinetics were observed. For example, in *YAHI*, the increase in lipid content was more progressive, remaining significant during the deceleration phase (from 5.1 to 7.2% of CDW) whereas the increase was less pronounced for *YAAL* (from 4.0 to 4.5% of CDW). There were two exceptions to this rule: *YALI*, the lipid content of which remained stable over time, and *YAYA*, the lipid content of which decreased gradually from 6 to 4.7% of CDW.

**Figure 5:**
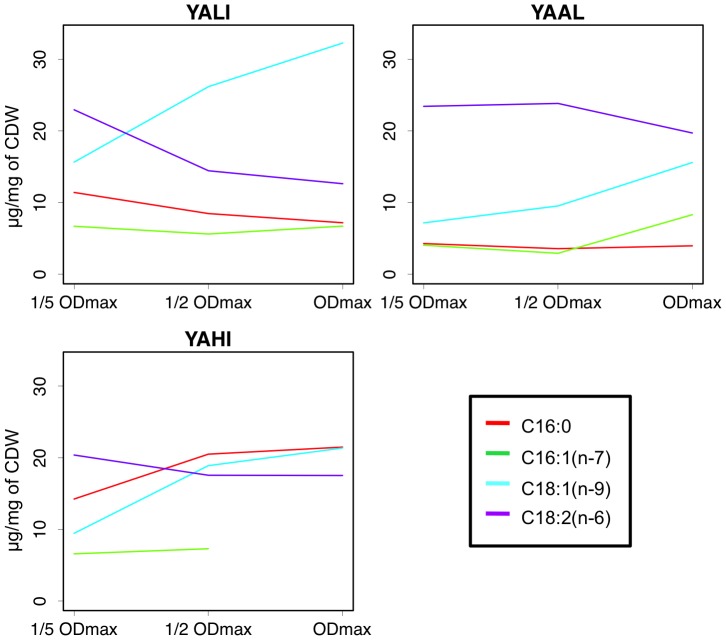
Changes in cellular lipid composition over time in strains grown on glucose media. *YALI*: *Yarrowia lipolytica* W29, *YAAL*: *C. alimentaria* CBS10151, *YAHI*: *C. hispaniensis* CBS9996. Quantities are in mg/g CDW.

In most strains, C18:1(n-9) was the major compound that accumulated, its levels increasing over the entire time course. However, in *YAAL*, *YAYA* and *YAHI*, this compound was not the principal compound at OD max. In *YAAL* and *YAYA*, C18:2(n-6) was the major compound at all three time points considered. In *YAHI*, the increase in lipid content was due to both C16:0 and C18:1(n-9) (Figure 5, Additional Fig S5, Additional Table S5).

## Discussion

We analyzed eight closely related species of the *Yarrowia* clade, to obtain preliminary data concerning their physiology and, in particular, their ability to grow on and to assimilate HS, before beginning comparative genomic and transcriptomic studies. All the data discussed below were obtained from a single isolate per species and may, therefore, not be representative of the entire species.

As expected on the basis of published findings, despite their isolation from highly diverse environments (Table 1), these strains displayed a very limited range of non hydrophobic substrate used, with low levels of variability[18], [19], [20], [21]. Glucose, fructose and glycerol were the only non hydrophobic carbon sources used by all strains of the clade. The screening of these strains for the detection of enzymatic activities useful for the exploitation of agricultural by-products, such as xylose degradation, does not therefore appear to be pertinent.

As *Y. lipolytica* uses HS efficiently, we tested the eight strains on 13 different substrates (alkanes, fatty acids and their methylated derivatives, and triglycerides) with carbon chains of different lengths (C4 to C22). Most of these substrates had never before been tested as substrates for species of this clade. We observed differences in growth on these substrates, the size of the hydrolysis halo and sensitivity to short-chain fatty acids between the species tested. These fatty acids were toxic to all strains, but *YAPH* and *YAHI* were able to grow on YP in the presence of C6 after a very long time lag.

Toxicity was clearly observed for *YALI* on MMB-methyldecanoate, but not on YP methyldecanoate (Figure 2). This may result from the balance between transport and degradation on the one hand and the hydrolysis of methyl-C10 by lipases/esterases to release decanoate, a compound toxic to cells, on the other. At high cell density, methyl-C10 hydrolysis by lipases may occur too rapidly, releasing too much toxic C10 for the cells to cope with, whereas, at lower cell density, the release of toxic C10 might be slow enough to prevent cell intoxication but permit cell growth. The halo was smaller for *YAPH* on the same medium, which thus released C10 at a rate compatible with growth. On YP supplemented with methyl-C10, both strains grew vigorously. All strains of the *Yarrowia* clade were able to grow efficiently on YP in the absence of any other carbon source (see additional Figure S2). On YP-methyl decanoate, no hydrolysis halo was observed with *YALI*, suggesting that the corresponding lipase/esterase is repressed in the presence of peptone. This putative repression was not observed with *YAPH*, suggesting that cell intoxication was prevented either by slow C10 uptake or by the rapid β-oxidation of C10. Further studies involving comparative genomics and transcriptomics would make it possible to test these hypotheses. These approaches might also shed light on the evolution of the *POX* and *LIP* gene networks in *YAPH* and *YALI*, in which six different acyl-CoA oxidases (Pox1p to Pox6p) of different chain length specificities have been described [40], [41], [42], together with 16 lipases and four esterases with different patterns of regulation and chain-length specificities [7].

We tried to determine whether these yeasts were oleaginous, by analyzing lipid accumulation and synthesis. However, the concept of what constitutes an oleaginous yeast remains unclear. Some authors consider that microorganisms should be able to accumulate more than 20% of CDW in the form of lipid to be considered oleaginous, whereas others consider that oleaginous organisms should be able to synthesize lipids from non HS carbon sources and to store them [8]. This is a crucial difference as some species are probably able to synthesize, but not to accumulate lipids efficiently, whereas other species may only accumulate lipids from the extracellular medium efficiently. These two capacities should thus be estimated independently, on different carbon sources, such as glucose for lipid synthesis and a model oleic acid medium previously used for *YALI* for the assessment of storage capacities [16].

Our results demonstrated that all strains cultured on oleic acid accumulated more than 30% of their CDW in the form of lipid at least one time point. Our findings suggest that all species of the clade are oleaginous. Most accumulated smaller amounts of lipid than *YALI*, but at least one, *YAHI*, accumulated almost 30% more lipid than *YALI*. However, different accumulation profiles were observed during the growth phase: some strains progressively accumulated triglycerides (*YAOS* and *YAPH*), others remobilized lipids (*YADE*, *YAGA* and *YAHO*), and lipid content remained essentially stable in *YALI* and *YAAL*. Lower and less variable levels of lipid accumulation were observed on glucose. Depending on the strain, the major compound accumulated was C18:1, C18:2 or C16:0. No molecules with a longer chain length were observed, by contrast to what has been reported for *Saccharomyces cerevisiae* and *Hansenula polymorpha* [43], [44]. *Candida albicans* can synthesize and accumulate C18:3 to levels of up to 22.7% of total fatty acids [43], but none of the yeasts of the *Yarrowia* clade was able to synthesize C18:3. This finding is consistent with the absence from the nuclear genomes of *YAGA, YAYA, YAAL, YAPH* and *YAHI* of a gene encoding a Δ15-desaturase, whereas homologs of YALI0C05951g (Δ9-desaturase) and YALI0B10153g (Δ12-desaturase) were identified (unpublished data). The variable levels of the various compounds may reflect differences in the activity or expression of the Δ12-desaturase (for *YAAL*, *YAYA*) or elongases (for *YAHI*).

The major conclusion of this work is that all species of the *Yarrowia* clade are oleaginous, but that they differ in their profiles of HS use and lipid accumulation. These data should facilitate the development of more robust, better performing strains for lipid accumulation and for fatty acid profile modification. Our findings also suggest that there are metabolic and genetic differences between the strains of the clade studied here, possibly reflecting species-specific differences in gene content and/or regulation. *C. hispaniensis*, which grew well on diverse substrates and displayed a high storage capacity, appears to be a promising model for biotechnological applications *per se* or as a source of genes for the improvement of *Y. lipolytica*. Further comparative genomic and transcriptomic studies are underway in the *Yarrowia* clade, to establish correlations with phenotypic variability and to obtain evidence for their genetic bases.

## Supporting Information

Figure S1 **Growth curves of YAAL on YPD at different temperatures.** The growth curves at 15°C, 21°C and 25°C are shown as blue squares, green dots, and red triangles, respectively.
(PDF)

Figure S2 **Drop tests on YP (A) and MMB (B) media with alkanes and methyl-esters of various chain lengths.** Only one spot is presented for strains *YALI*, *YADE*, *YAGA*, *YAYA* and *YAOS*. Strains are represented by an abbreviation of their species name, as shown in Table 1.
(PDF)

Figure S3 **Growth curves established on 2% oleic acid (A) and 2% glucose (B) media for the strains of the** ***Yarrowia*** **clade, over a period of 50 h.** Strain names are abbreviated as follows: *YALI* (*Y. lipolytica* W29), *YAYA* (*Y. yakushimensis* CBS10253), *YADE (Y. deformans* CBS2071), *YAGA (C. galli* CBS9722), *YAOS (C. oslonensis* CBS10146), *YAHO (C. hollandica* CBS4855), *YAPH (C. phangngensis* CBS10407), *YAAL (C. alimentaria* CBS10151), *YAHI (C. hispaniensis* CBS9996).
(PDF)

Figure S4 **Growth curve and time points used for** ***YAGA*** **cultured on glucose (2%).** The experimental growth curve is shown in red. Horizontal dotted lines correspond to the maximum OD, 1/2 OD max and 1/5 of OD max. Circles, triangles and diamonds indicate the OD at the time points used for lipid accumulation tests, at 4.6 h, 11.6 h and 23.7 h of culture, respectively.
(PDF)

Figure S5 **Changes in cellular lipid composition over time for strains grown on oleic acid (A) and glucose (B).** Quantities are in mg/g CDW. For each species, three different physiological states are represented: 1/5 the OD max (1/5), 1/2 the OD max (1/2) and the OD max (max).
(PDF)

Table S1 **Growth capacities of the species of the** ***Yarrowia*** **clade on non hydrophobic carbon.**
(XLSX)

Table S2 **Primers used to amplify the protein-coding genes.**
(XLSX)

Table S3 **Presence of a halo on solid hydrophobic media.**
(XLSX)

Table S4 **Characteristics of growth over time for cultures on oleic acid and glucose media: time points, optical density, lipid content and cell dry weight.**
(XLSX)

Table S5 **Profile of fatty acid accumulation (% of CDW) on glucose and oleic acid media.**
(XLS)
