# Supplementary material for: Comparative Physiology of Oleaginous Species from the Yarrowia Clade
Source: PLoS One. 2013 May 7;8(5):e63356. doi: 10.1371/journal.pone.0063356 (PMC3646758; doi:10.1371/journal.pone.0063356)
Supplement: Figure S1 — Growth curves of YAAL on YPD at different temperatures. The growth curves at 15°C, 21°C and 25°C are shown as blue squares, green dots, and red triangles, respectively. (PDF) [file pone.0063356.s001.pdf]

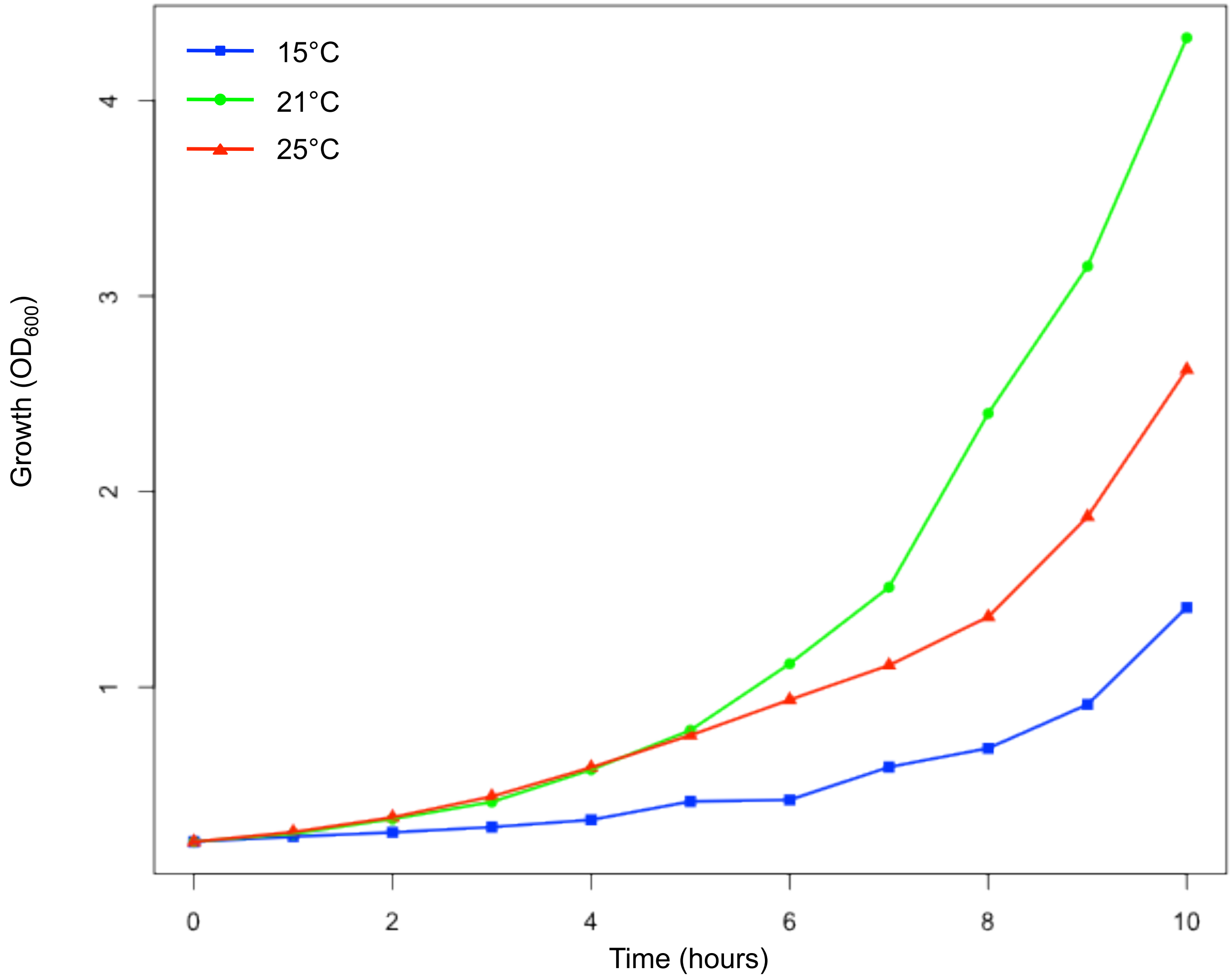

Additional Figure S1: Growth curves of YAAL on YPD at different temperatures. The growth curves at 15°C, 21°C and 25°C are represented in blue with squares, in green with dots, and in red with triangles, respectively.
