# Supplementary material for: Comparative Physiology of Oleaginous Species from the Yarrowia Clade
Source: PLoS One. 2013 May 7;8(5):e63356. doi: 10.1371/journal.pone.0063356 (PMC3646758; doi:10.1371/journal.pone.0063356)
Supplement: Figure S2 — Drop tests on YP (A) and MMB (B) media with alkanes and methyl-esters of various chain lengths. Only one spot is presented for strains YALI, YADE, YAGA, YAYA and YAOS. Strains are represented by an abbreviation of their species name, as shown in Table 1. (PDF) [file pone.0063356.s002.pdf]

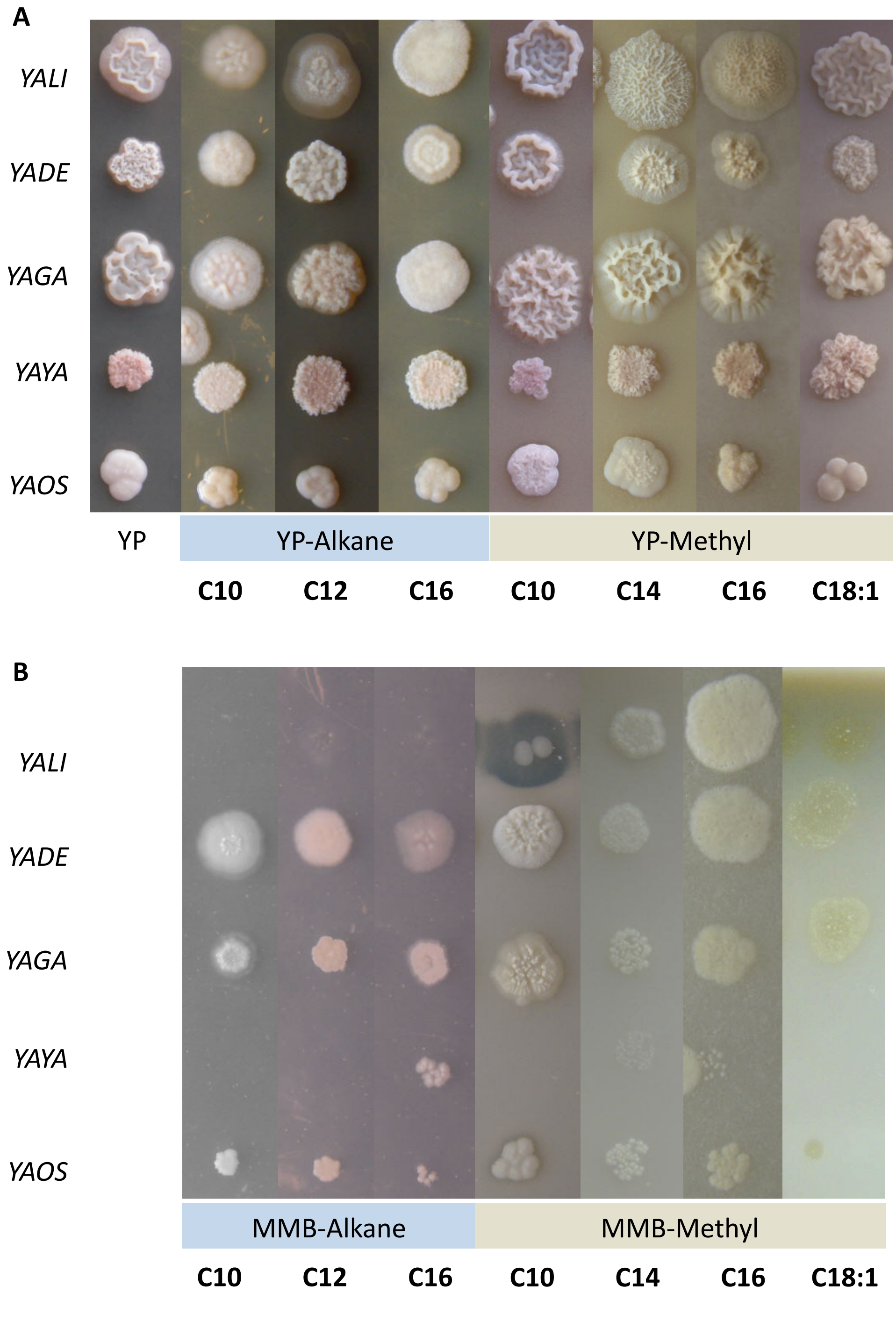

Additional Figure S2: Drop tests on YP (A) and MMB (B) media with alkanes and methyl-esters of different chain length. Only one spot is presented for strains *YALI*, *YADE*, *YAGA*, *YAYA* and *YAOS*. Strains are represented by an abbreviation of their species name as shown in Table 1.
