# Supplementary material for: Comparative Physiology of Oleaginous Species from the Yarrowia Clade
Source: PLoS One. 2013 May 7;8(5):e63356. doi: 10.1371/journal.pone.0063356 (PMC3646758; doi:10.1371/journal.pone.0063356)
Supplement: Figure S3 — Growth curves established on 2% oleic acid (A) and 2% glucose (B) media for the strains of the Yarrowia clade, over a period of 50 h. Strain names are abbreviated as follows: YALI (Y. lipolytica W29), YAYA (Y. yakushimensis CBS10253), YADE (Y. deformans CBS2071), YAGA (C. galli CBS9722), YAOS (C. oslonensis CBS10146), YAHO (C. hollandica CBS4855), YAPH (C. phangngensis CBS10407), YAAL (C. alimentaria CBS10151), YAHI (C. hispaniensis CBS9996). (PDF) [file pone.0063356.s003.pdf]

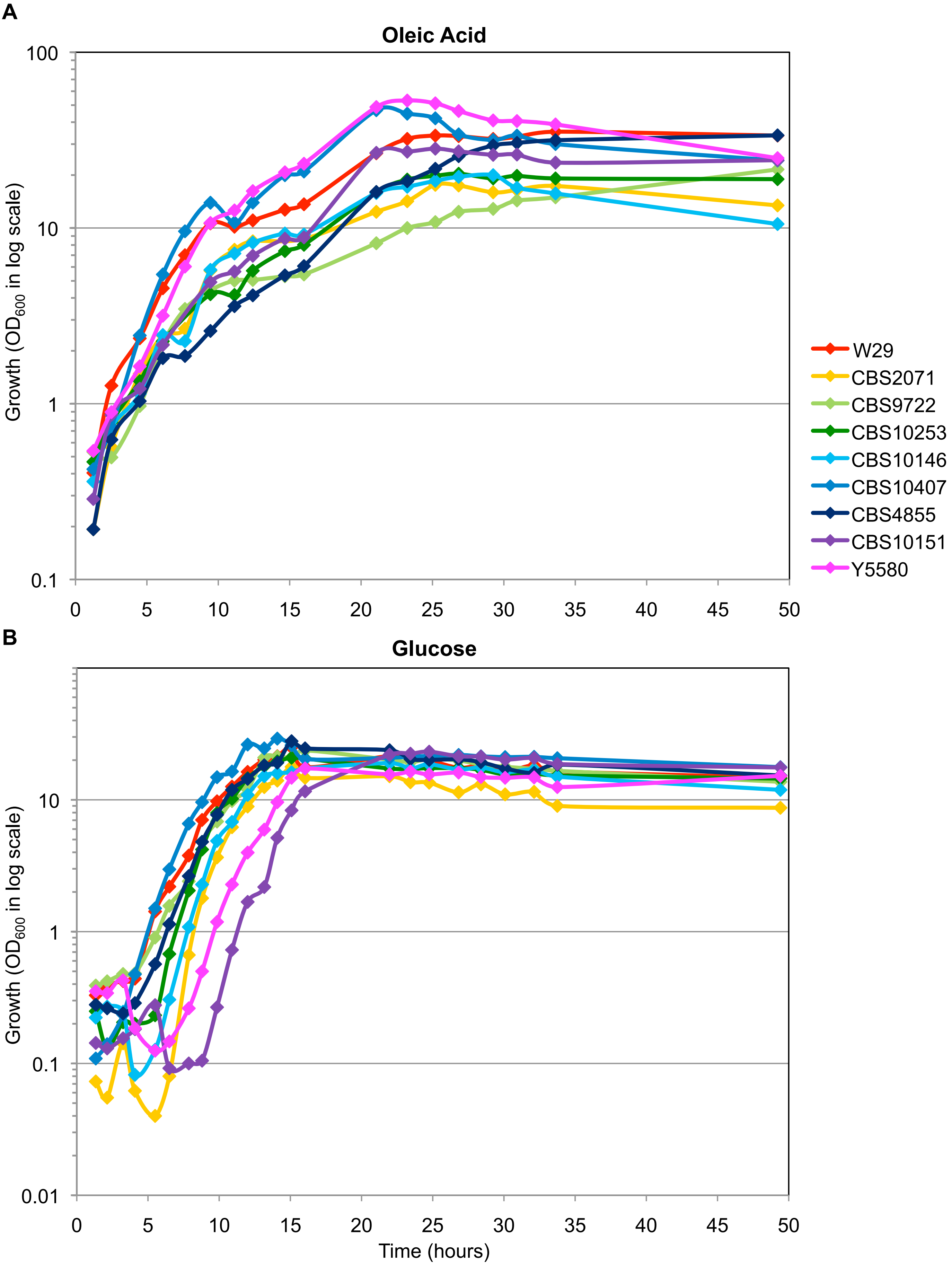

Additional Figure S3: Growth curves established on oleic acid 2% (A) and glucose 2% (B) media for the strains of the *Yarrowia* clade during 50 h. Strain names are abbreviated as follows: *YALI* (*Y. lipolytica* W29), *YAYA* (*C. yakushimensis* CBS10253), *YADE (C. deformans* CBS2071), *YAGA (C. galli* CBS9722), *YAOS (C. oslonensis* CBS10146), *YAHO (C. hollandica* CBS4855), *YAPH (C. phangngensis* CBS10407), *YAAL (C. alimentaria* CBS10151), *YAHI (C. hispaniensis* CBS9996).
