# Supplementary material for: Comparative Physiology of Oleaginous Species from the Yarrowia Clade
Source: PLoS One. 2013 May 7;8(5):e63356. doi: 10.1371/journal.pone.0063356 (PMC3646758; doi:10.1371/journal.pone.0063356)
Supplement: Figure S4 — Growth curve and time points used for YAGA cultured on glucose (2%). The experimental growth curve is shown in red. Horizontal dotted lines correspond to the maximum OD, 1/2 OD max and 1/5 of OD max. Circles, triangles and diamonds indicate the OD at the time points used for lipid accumulation tests, at 4.6 h, 11.6 h and 23.7 h of culture, respectively. (PDF) [file pone.0063356.s004.pdf]

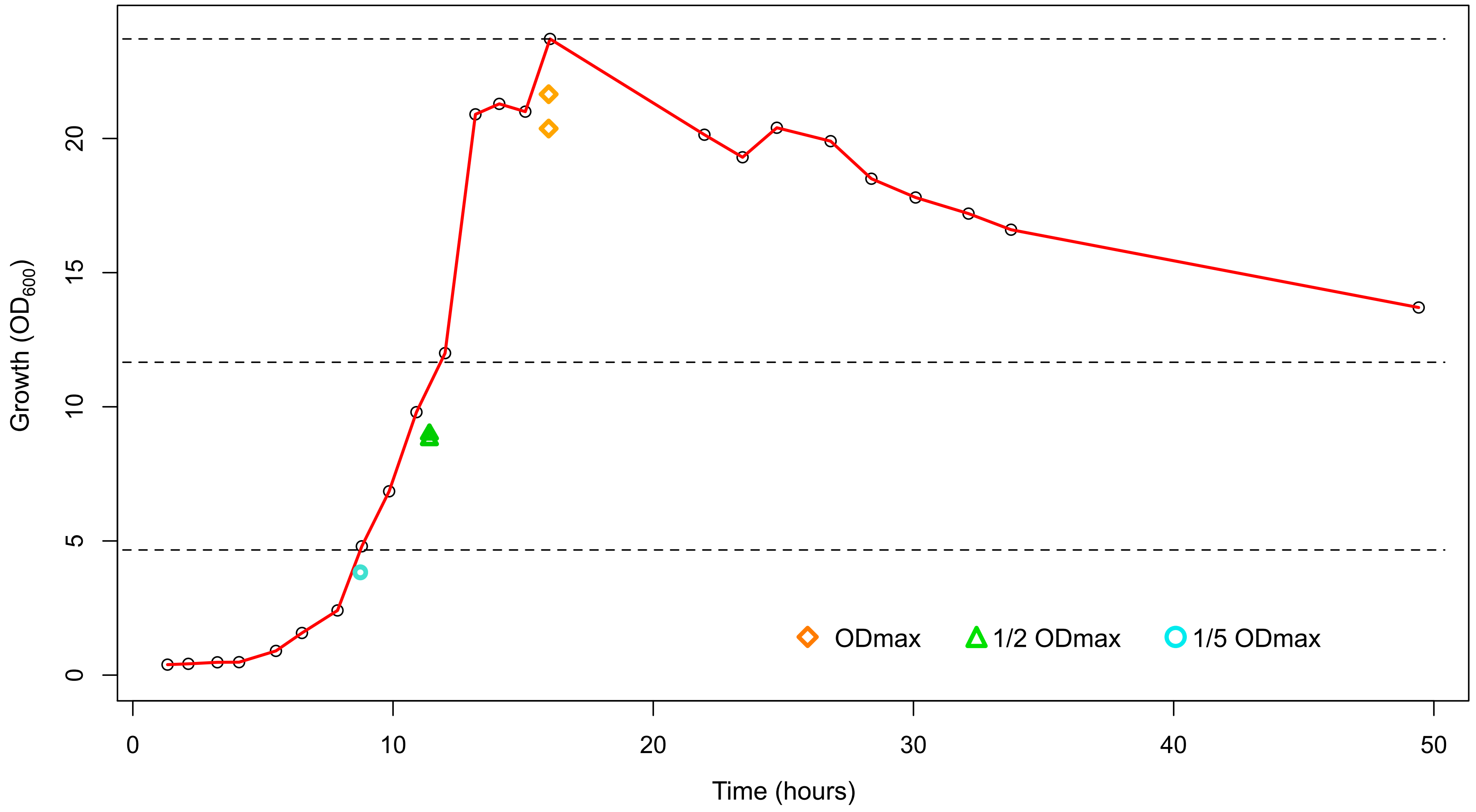

Additional Figure S4: Growth curve and time points used for *YAGA* cultivated on Glucose (2%). Experimental growth curve is in red. Horizontal dotted lines correspond to the maximum OD, to the 1/2 and to the 1/5 of OD max. Circles, triangles and diamonds indicate OD at the time points used for lipid accumulation tests at 4.6 h, 11.6 h and 23.7 h of culture, respectively.
