# Supplementary material for: Comparative Physiology of Oleaginous Species from the Yarrowia Clade
Source: PLoS One. 2013 May 7;8(5):e63356. doi: 10.1371/journal.pone.0063356 (PMC3646758; doi:10.1371/journal.pone.0063356)
Supplement: Figure S5 — Changes in cellular lipid composition over time for strains grown on oleic acid (A) and glucose (B). Quantities are in mg/g CDW. For each species, three different physiological states are represented: 1/5 the OD max (1/5), 1/2 the OD max (1/2) and the OD max (max). (PDF) [file pone.0063356.s005.pdf]

A

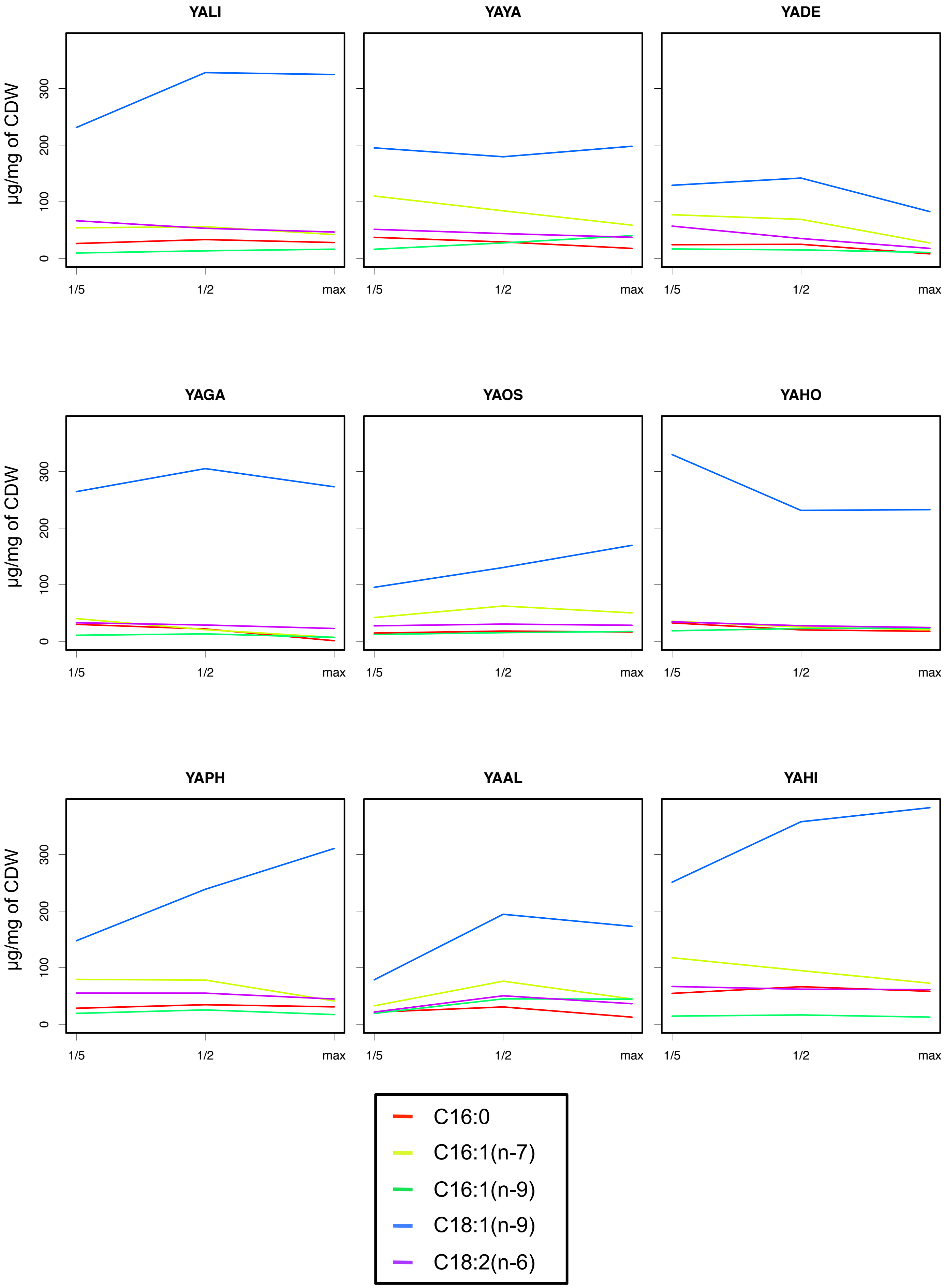
YALI
YAYA
YADE
YAGA
YAOS
YAHO
YAPH
YAAL
YAHI
µg/mg of CDW
0
100
200
300
1/5
1/2
max
C16:0
C16:1(n-7)
C16:1(n-9)
C18:1(n-9)
C18:2(n-6)

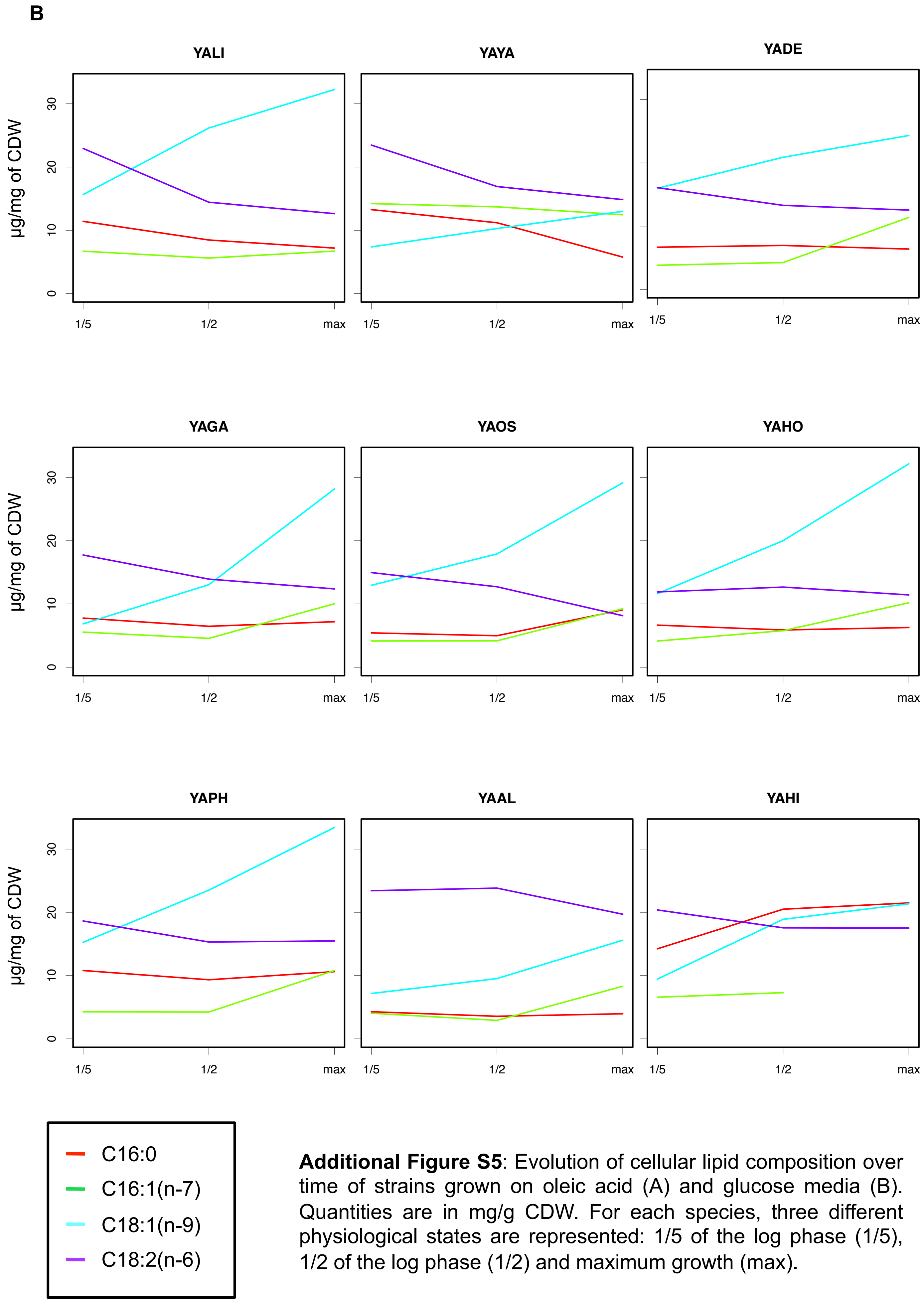

Additional Figure S5: Evolution of cellular lipid composition over time of strains grown on oleic acid (A) and glucose media (B). Quantities are in mg/g CDW. For each species, three different physiological states are represented: 1/5 of the log phase (1/5), 1/2 of the log phase (1/2) and maximum growth (max).
